# The Effect of the Interaction between Obesity and Drinking  on Hyperuricemia in Japanese Male Office Workers

**DOI:** 10.2188/jea.JE20080016

**Published:** 2009-01-30

**Authors:** Hiroshi Shiraishi, Hiroshi Une

**Affiliations:** 1 Department of Rehabilitation Medicine, Imazu Red Cross Hospital, Fukuoka, Japan; 2 Department of Hygiene and Preventive Medicine, School of Medicine, Fukuoka University, Fukuoka, Japan

**Keywords:** drinking, epidemiology, hyperuricemia, interaction, obesity

## Abstract

**Background:**

Obesity and drinking are acknowledged risk factors for hyperuricemia. However, the effect of the interaction between obesity and drinking on hyperuricemia is not well understood.

**Methods:**

The cases comprised 3028 male office workers with hyperuricemia (serum uric acid level ≥7.0 mg/dL); the controls were 5348 men with a serum uric acid level less than 6.0 mg/dL. Logistic regression analysis was used to assess risk factors for hyperuricemia and the interaction between obesity and drinking in hyperuricemia. Participants were divided into 2 groups according to their BMI: individuals with a BMI of 25 or higher were classified as obese and those with a BMI less than 25 were classified as non-obese. In addition, participants were divided into 5 groups based upon their ethanol consumption. The 2 variables were then combined to create 10 groups.

**Results:**

With non-obese non-drinkers as the reference category, the odds ratio for hyperuricemia was 1.80 for non-obese drinkers of less than 25 mL/day of ethanol, 2.15 for non-obese drinkers of 25–49 mL/day, 2.60 for non-obese drinkers of 50–74 mL/day, 2.56 for non-obese drinkers of 75+ mL/day, 4.40 for obese non-drinkers, 5.74 for obese drinkers of less than 25 mL/day, 6.57 for obese drinkers of 25–49 mL/day, 5.55 for obese drinkers of 50–74 mL/day, and 7.77 for obese drinkers of 75+ mL/day. The interaction between obesity and drinking in hyperuricemia was statistically significant.

**Conclusion:**

Our results suggest that although combining the effects of obesity and drinking did not result in a multiplicative increase in the risk for hyperuricemia, the combined risk was greater than the sum of the effects of obesity and drinking.

## INTRODUCTION

The lifestyle of Japanese has become progressively more westernized, and both fat intake and alcohol consumption have increased greatly.^1^ The prevalence of hyperuricemia in Japanese men increased from 5% in the 1960s to 20% in the 1990s.^2^ Hyperuricemia is related not only to an increased risk of gout, but also to an increased risk of cardiovascular diseases such as stroke and ischemic heart disease. A positive association between serum uric acid and cardiovascular diseases has been recognized since the 1950s^3^ and has been confirmed by many epidemiological studies.^4^^–^^6^ Although it is unclear whether hyperuricemia plays an independent causal role in cardiovascular disease, increased serum uric acid levels are linked to obesity, hypertension, glucose intolerance, and dyslipidemia, all of which are associated with an increased risk for cardiovascular disease.^7^^,^^8^

Obesity and alcohol consumption are known determinants of serum uric acid level, and obesity and serum uric acid level correlate positively regardless of age or sex. Serum uric acid level also decreases with weight loss.^9^ A positive dose-response relationship was found between alcohol consumption and serum uric acid levels. Compared with participants who did not drink alcohol, the odds ratio (OR) for hyperuricemia was 2.89 for participants who consumed at least 50 mL/day of ethanol.^10^

Although obesity and drinking—which are both closely connected with lifestyle—are risk factors for hyperuricemia, the importance of the interaction between obesity and drinking has not been clarified. We therefore examined the effect of the interaction between obesity and drinking on hyperuricemia.

## METHODS

### Participants

A cross-sectional survey was performed using data from health checkups of male employees of a large company in western Japan. We enrolled 12,937 men aged 20 to 69 years in 2003. All participants underwent annual health checkups including blood chemistry analysis and a physical examination. We excluded 483 men who were under treatment for hypertension or gout and those with abnormal renal function (serum creatinine ≥1.5 mg/dL). Of the remaining 12,454 men, 3028 had a serum uric acid level of 7.0 mg/dL or higher, 4078 had a serum uric acid level of 6.0–6.9 mg/dL, and 5348 had a serum uric acid level of less than 6.0 mg/dL. We then analyzed the 3028 men with hyperuricemia (serum uric acid level of ≥7.0 mg/dL) as cases and the 5348 men with a serum uric acid level less than 6.0 mg/dL as controls. The Japanese Society of Gout and Nucleic Acid Metabolism defines hyperuricemia as a serum uric acid level of 7.0 mg/dL or more and recommends maintaining the serum uric acid level at less than 6.0 mg/dL.^2^

Informed consent was obtained from each participant. Identifying data such as name and full birth date were not used in the analysis. The study protocol was approved by the Ethics Committee of Fukuoka University.

### Methods

The survey included a questionnaire, physical examination, and collection of blood samples for laboratory analysis. A questionnaire was used to interview participants about their lifestyle, including alcohol consumption and smoking habits. Questions about alcohol intake concerned the type of alcoholic beverages consumed, the weekly frequency of alcohol consumption, and the usual amount of alcohol consumed daily. Average daily ethanol consumption was calculated based on the type of alcoholic beverages consumed, the weekly frequency of alcohol consumption, and the usual amount of alcohol consumed daily. We calculated the units of ethanol for the average daily intake of each type of beverage consumed. Average daily alcohol consumption was calculated assuming that 180 mL (1 unit) of Japanese sake, 633 mL (1 bottle) of beer, and 100 mL (1 unit) of *shochu* each contain 25 mL of ethanol. Participants were classified into 5 categories according to the quantity consumed: non-drinkers, those drinking less than 25 mL per day (<25 mL/day), 25 mL to less than 50 mL per day (25–49 mL/day), 50 mL to less than 75 mL per day (50–74 mL/day), and 75 mL or more per day (≥75 mL/day).

Physical examination included height and body weight measurements. The body mass index (BMI) was calculated as weight in kilograms divided by height in meters squared. Participants were divided into 2 groups according to their BMI: those with a BMI ≥25 were classified as obese, and those with a BMI <25 as non-obese. We then combined the 2 variables of BMI and drinking to create 10 groups.

Smoking habits were classified into 2 categories: ever-smokers (ie, ex-smokers and current smokers) and never-smokers.

Venous blood was taken for serum biochemical measurement after an overnight fast. Serum uric acid concentration was determined with an auto analyzer (Hitachi 7350) by the uricase method.

### Statistical analysis

Logistic regression analysis was performed to assess risk factors for hyperuricemia. ORs together with 95% confidence intervals (CI) and corresponding *P* values for all factors were calculated from multivariate models adjusted for age. The interaction between obesity and drinking in hyperuricemia was investigated by adding an interaction term into the model, and their significance was ascertained. Statistical analyses were conducted using SAS statistical software package Version 9.1 (SAS Institute Inc.).

## RESULTS

### Analysis of risk factors for hyperuricemia

Table 1 shows the age-adjusted ORs for hyperuricemia for different ages, BMI, alcohol intake, and smoking status. When individuals with a BMI of less than 22 were defined as the reference group, the OR for hyperuricemia was 2.13 for those with a BMI of 22.0–24.9, and 5.07 for those with a BMI of 25 or more. With non-drinkers as the reference group, the OR for hyperuricemia was 1.56 for those who consumed less than 25 mL/day of ethanol, 1.80 for 25–49 mL/day of ethanol, 1.95 for 50–74 mL/day of ethanol, and 2.17 for 75+ mL/day of ethanol. For all categories of BMI and alcohol intake, the differences in the ORs for hyperuricemia were statistically significant (*P* < 0.001). With never-smokers as the reference category, the OR for hyperuricemia was 0.97 for ever-smokers; smoking status was not associated with a risk for hyperuricemia (*P* = 0.52). With respect to age, the highest risk was observed in the 30–39 age group.

**Table 1.** Odds ratios for hyperuricemia (≥7.0 vs. <6.0 mg/dL)

Variable	Cases (7.0+ mg/dL)	Controls (<6.0 mg/dL)	Total	Odds ratios (95% CI) *	*P*
Age (years)					
20–29	1142 (70%)	483 (30%)	1625 (100%)	1.00 (reference)	
30–39	1861 (62%)	1124 (38%)	2985 (100%)	1.15 (1.00–1.32)	0.051
40–49	1320 (60%)	869 (40%)	2189 (100%)	1.11 (0.96–1.29)	0.153
50–59	931 (64%)	514 (36%)	1445 (100%)	0.94 (0.80–1.10)	0.424
60–69	94 (71%)	38 (29%)	132 (100%)	0.67 (0.44–1.01)	0.056
Body mass index (kg/m^2^)					
<22.0	554 (20%)	2230 (80%)	2784 (100%)	1.00 (reference)	
22.0–24.9	1146 (36%)	2090 (64%)	3236 (100%)	2.13 (1.89–2.40)	<.0001
25.0+	1328 (56%)	1028 (46%)	2356 (100%)	5.07 (4.47–5.74)	<.0001
Alcohol intake					
Non-drinker	566 (27%)	1538 (73%)	2104 (100%)	1.00 (reference)	
Daily ethanol consumption (mL)					
<25	1188 (37%)	2060 (63%)	3248 (100%)	1.56 (1.38–1.77)	<.0001
25–49	735 (41%)	1064 (59%)	1799 (100%)	1.80 (1.56–2.08)	<.0001
50–74	360 (43%)	475 (57%)	835 (100%)	1.95 (1.63–2.33)	<.0001
75+	179 (46%)	211 (54%)	390 (100%)	2.17 (1.71–2.74)	<.0001
Smoking status					
Never-smoker	1327 (36%)	2393 (64%)	3720 (100%)	1.00 (reference)	
Ever-smoker	1701 (37%)	2955 (63%)	4656 (100%)	0.97 (0.88–1.07)	0.509

*: Adjusted for variables in table

CI: Confidence Interval

### Analysis of the interactions between obesity and drinking in hyperuricemia

Table 2 shows age-adjusted ORs for hyperuricemia for the 10 different groups created by combining the variables of BMI and drinking. With non-obese non-drinkers as the reference category, the ORs were 1.80 for non-obese drinkers of <25 mL/day, 2.15 for non-obese drinkers of 25–49 mL/day, 2.60 for non-obese drinkers of 50–74 mL/day, 2.56 for non-obese drinkers of 75+ mL/day, 4.40 for obese non-drinkers, 5.74 for obese drinkers of <25 mL/day, 6.57 for obese drinkers of 25–49 mL/day, 5.55 for obese drinkers of 50–74 mL/day, and 7.77 for obese drinkers of 75+ mL/day. Statistical significance was observed in all categories (*P* < 0.001).

**Table 2.** Odds ratios for the interaction between obesity and drinking in hyperuricemia (≥7.0 vs. <6.0 mg/dL)

Variables	Cases (7.0+ mg/dL)	Controls (<6.0 mg/dL)	Odds ratios (95% CI) *	*P*
Non-obese, Non-drinker	285	1,259	1.00 (reference)	
Non-obese, Ethanol consumption <25 mL/day	694	1,689	1.80 (1.54–2.10)	<.0001
Non-obese, Ethanol consumption 25–49 mL/day	416	852	2.15 (1.80–2.56)	<.0001
Non-obese, Ethanol consumption 50–74 mL/day	211	357	2.60 (2.09–3.22)	<.0001
Non-obese, Ethanol consumption 75+ mL/day	94	163	2.56 (1.92–3.42)	<.0001
Obese, Non-drinker	281	279	4.40 (3.57–5.43)	<.0001
Obese, Ethanol consumption <25 mL/day	494	371	5.74 (4.76–6.92)	<.0001
Obese, Ethanol consumption 25–49 mL/day	319	212	6.57 (5.28–8.17)	<.0001
Obese, Ethanol consumption 50–74 mL/day	149	118	5.55 (4.21–7.32)	<.0001
Obese, Ethanol consumption 75+ mL/day	85	48	7.77 (5.32–11.36)	<.0001

*: Adjusted for age and variables in table

Obese: Body mass index ≥25 kg/m^2^; Non-obese: Body mass index <25 kg/m^2^

CI: Confidence Interval

The interactions between obesity and drinking in hyperuricemia were statistically significant (*P* < 0.001), and the combined effects of obesity and drinking on hyperuricemia were greater than their sum, except for obese drinkers of 50–74 mL/day.

## DISCUSSION

In this study using data from health checkups, the interaction between obesity and drinking in hyperuricemia was found to be statistically significant. To our knowledge, this is the first report to document an interaction between obesity and drinking in hyperuricemia in Japanese male workers.

Many, but not all, studies have found that hyperuricemia is an independent risk factor for cardiovascular disease.^4^^–^^6^^,^^11^^–^^13^ Several studies did not show an independent association after adjustment for concomitant risk factors for cardiovascular disease.^14^^–^^16^ Thus, the specific role of serum uric acid in the development of cardiovascular disease remains uncertain. Recently, it has been reported that uric acid, under both in vitro and in vivo conditions, directly impairs the function of the vascular endothelium and stimulates the proliferation of vascular smooth muscle cells.^17^

Obesity and alcohol intake are lifestyle characteristics related to hyperuricemia. Positive correlations between serum uric acid level and both obesity and alcohol intake have been reported in many epidemiological studies.^18^^–^^20^ The present study has also showed that the OR for hyperuricemia increases with an increase in BMI or alcohol intake.

Alcohol intake has been identified as an important risk factor for hyperuricemia in a number of previous studies.^21^^–^^23^ Specifically, alcohol intake stimulates ethanol metabolism in the liver, accompanied by increased purine catabolism, which ultimately leads to a surge in the production of uric acid as a metabolite.^21^^,^^24^ In addition, serum uric acid level rises as a result of poor lactic acid excretion in the liver, which is a result of increased ethanol metabolism.^25^^,^^26^ Alcoholic drinks themselves contain abundant amounts of purine, which increases the load on the system. In particular, beer contains ten times more purine than other alcoholic drinks. Yamamoto et al found that beer tends to increase serum uric acid level more effectively than other alcoholic beverages.^27^ However, Sugie et al found that risk of hyperuricemia did not depend on the type of alcoholic beverage. There remains disagreement regarding the risk for hyperuricemia posed by different types of alcoholic beverages.^10^

As for the link between obesity and hyperuricemia, our study found that the OR for hyperuricemia increased in relation to the degree of obesity. The mechanism underlying this obesity-linked increase in serum uric acid level involves 2 factors: excessive uric acid production and poor uric acid excretion. Matsuura et al studied the relation between obesity type and hyperuricemia, and found that subcutaneous fat-type obesity is primarily related to poor uric acid excretion, while visceral fat-type obesity is primarily related to excessive uric acid production,^28^ which may occur because visceral fat accumulation induces excessive uric acid production, which in turn leads to an elevated influx of plasma free fatty acid into the portal vein and liver, stimulation of neutral fat synthesis, and a consequent attendant surge in uric acid production in the activated uric acid synthesis pathway.^29^^,^^30^ In the case of poor uric acid excretion, insulin resistance or hyperinsulinemia is believed to cause alterations in the transport of uric acid (a substrate co-transported with sodium) in the renal tubules.^31^ In obesity-linked insulin resistance, uric acid clearance becomes inefficient, which leads to functional impairments of uric acid excretion.^32^^,^^33^

In the present study, the interaction between obesity and drinking in hyperuricemia was statistically significant. Although the combined effects of obesity and drinking did not multiplicatively increase the odds of hyperuricemia, the combined risk was greater than the sum of the effects of obesity and drinking. These results indicate that obese men need to avoid heavy drinking. Regarding the impact of different risk factors on hyperuricemia, we found that the OR of hyperuricemia attributable to obesity was higher than that attributable to alcohol intake. Therefore, lifestyle changes to control body weight effectively are very important in preventing hyperuricemia. However, because the OR for hyperuricemia in non-obese men who drank moderately was relatively low, regulating moderate alcohol intake is undesirable, as it may have a negative effect on the risk for cardiovascular disease, as well as adversely affect social life, because moderate alcohol intake has been found to reduce the risk of cardiovascular disease.^34^^–^^36^

This study has certain limitations. First, it is a cross-sectional study, so no temporal relationship between hyperuricemia on one hand, and obesity and drinking on the other, can be inferred. However, it can be assumed that drinking habits and obesity remain relatively unchanged over a long period of time, and therefore are likely to be present before the development of hyperuricemia. Second, this study did not consider nutritional status, which is closely related to hyperuricemia, and instead used BMI as an indicator of nutritional status. The need remains for a prospective study of hyperuricemia with more detailed information on nutritional status.
